# Neurological Complications Associated With Varicella-Zoster Virus Infection

**DOI:** 10.7759/cureus.72305

**Published:** 2024-10-24

**Authors:** André Assunção, Lucinda Amorim-Delgado, Catarina Barros-Azevede, Ângela Dias, Catarina Magalhães

**Affiliations:** 1 Department of Pediatrics, Local Healthcare Unit São João, Porto, PRT; 2 Department of Pediatrics, Local Healthcare Unit Alto Ave, Guimarães, PRT; 3 Department of Pediatrics: Pediatric Neurology, Local Healthcare Unit Alto Ave, Guimarães, PRT

**Keywords:** autoimmune encephalomyelitis, immune-related neurological complications, infectious encephalitis, varicella encephalitis, varicella-zoster virus

## Abstract

Varicella-zoster virus is a highly contagious infection that primarily affects children. It typically presents as a mild, self-limiting illness with a distinctive rash. However, severe complications can arise, with skin and soft tissue infections being the most common. While neurological complications are less frequent, they are particularly serious and may include conditions such as encephalitis, meningitis, and transient neurological deficits. We report a case of a six-year-old child with neurological complications related to varicella. Initially, the child presented with the typical rash, fever, headache, and nausea. He was discharged after unremarkable physical and bloodwork analysis. Thirty-six hours later, his condition deteriorated: worsened headaches, ataxic gait, central hypotonia, and dysarthric speech. His bloodwork and computed tomography scan were normal, but cerebrospinal fluid (CSF) analysis showed pleocytosis and elevated protein levels; viral polymerase chain reaction and autoantibody tests in CSF were negative. His diagnosis was autoimmune encephalitis related to an infectious process, and he was treated with acyclovir and methylprednisolone for several days before any improvement. At one-month follow-up, minor neurological deficits persisted, but substantial improvement was achieved with physiotherapy and speech therapy. Although varicella is typically mild, it can lead to severe complications. This case illustrates the diagnostic and therapeutic challenges associated with neurologically complicated varicella infections. It also highlights the importance of comprehensive evaluation and management, as well as the long period that may be required for improvement. The response to acyclovir was lacking, and the CSF was negative, suggesting an immune-mediated process, emphasizing the complexity of postinfectious encephalitis. This case highlights the need for vigilance in pediatric varicella infections, particularly concerning neurological complications. A thorough clinical assessment and laboratory work-up are crucial to better diagnosing and properly addressing these complications. Early intervention and comprehensive rehabilitation are key to improving outcomes in children affected by such complications.

## Introduction

Varicella, commonly known as chickenpox, is a viral infection caused by Varicella-zoster virus (VZV), an alpha-herpesvirus that ubiquitously affects children and adults [1-3]. VZV infection is very common during childhood and is transmitted via airborne particles or direct contact with fluid from vesicles, boasting a contagious rate of over 60% [1,2].

Typically, it presents as a mild and self-limited illness characterized by a distinctive rash [1,2,4]. However, varicella can pose significant risks, particularly to immunocompromised individuals and adults at higher risk of severe varicella [1,4]. Complications, although infrequent in healthy children, are reported by some authors to occur in up to 20% of cases and may include skin superinfections, pneumonia, and neurological complications, such as encephalitis [5-9]. Reports suggest that encephalitis may affect one in 50,000 cases of unvaccinated children [2]; other less common manifestations include aseptic meningitis, transient neurological deficits, myelitis, and vasculitis [5,10,11]. These disorders typically develop within one week of symptom onset and require hospitalization for comprehensive management and monitoring [1]. In this report, we present a case of a six-year-old child who developed severe neurological complications during VZV infection, shedding light on the potential gravity of such manifestations in pediatric patients.

## Case presentation

A previously healthy six-year-old male child was brought to the emergency department with complaints of headache, nausea, abdominal pain, and fever. His mother also noticed an unsteady gait when he was febrile. Over the previous five days, he had developed a vesicular rash consistent with chickenpox and had a fever for the first three days of the illness. He was 118 cm high and 22 kg (both in the 50th to 85th percentile according to World Health Organization growth charts), had a normal constitution, and was well hydrated and well nourished. His physical examination presented a rash compatible with chickenpox, and his neurological examination was unremarkable. The bloodwork analysis, including total leukocytes and differential counts, showed no abnormalities for his age. His symptoms improved after oral hydration, ondansetron, and antipyretic medication, and he was discharged home.

Thirty-six hours later, he returned to the hospital due to worsening symptoms: headaches unresponsive to oral analgesics, recurring fever, vomiting, and difficulty walking. His vitals were 108/60 mmHg and 90 bpm, he was afebrile (36.7°C), and his oxygen saturation was 98%. During examination, his neurological status fluctuated between full alertness and drowsiness, but even when awake, his speech was dysarthric and slurred, and he presented cephalic and trunk instability; he had dysmetria with tremor of upper limbs during movements, and he exhibited ataxic and unbalanced gait when walking. There were no other abnormalities in eye movements, muscle strength, reflexes, or his general physical examination. Blood tests and brain computed tomography showed no abnormalities. A lumbar puncture revealed pleocytosis (260 leucocytes/microliter, 99% mononuclear cells) and elevated protein levels (54.8 mg/dL) in the cerebral spinal fluid (CSF), but viral polymerase chain reaction (PCR) tests, including VZV, were negative. Based on the clinical picture and epidemiological context, the child was admitted to the hospital and started on acyclovir 1,500 mg/m^2^/day, which was maintained until the eighth day of hospitalization.

In the first 48 hours of admission, this condition worsened with increasing central hypotonia, worsened speech, and inability to walk or stand. He also developed dizziness, nausea, and vomiting triggered by movements and upper body instability, and, eventually, refusal to eat to avoid cephalic movements. On day 3 of admission, some psychomotor slowness ensued, and he did not seem to improve with acyclovir; methylprednisolone (30 mg/kg/day) was started for suspected postinfectious autoimmune encephalopathy, pending MRI and electroencephalogram results. We also further tested the previously collected CSF for autoantibodies (as anti-N-methyl-d-aspartate, anti-potassium channels, anti-gamma-aminobutyric acid, and glutamate). However, the results were negative; unfortunately, due to technical issues, antimyelin oligodendrocyte glycoprotein and aquaporin-4 autoantibodies were not assessed. MRI and electroencephalogram examinations were normal.

Over the next four days, the child's condition remained unchanged, with persistent refusal to eat or to move, requiring intravenous fluids at first and then enteral feeding by nasogastric tube. He also developed mild bradycardia (50-60 bpm while awake), maintaining his arterial blood pressure in the 50th to 95th percentile. Therefore, an electrocardiogram and cardiac ultrasound exams were requested, which revealed no cardiac abnormalities.

On the eighth day of hospitalization, gradual improvement began with reduced nausea and dizziness, and gradual recovery of central tonus motor function. Acyclovir and methylprednisolone were suspended, having completed a total of eight and five days, respectively. He was discharged on the 12th day with some residual neurological deficits, including mild gait imbalance and speech difficulties. At a one-month follow-up appointment, the child revealed some improvements but not completely. After starting physiotherapy and speech therapy, a full recovery was rapidly identified.

## Discussion

The presented case highlights the challenges in diagnosing and managing neurological complications related to VZV infection. Typically, this disease presents with mild and time-limited symptoms; however, in a few patients, neurological complications can develop, and they pose significant clinical dilemmas [1,2,5].

In our case, a six-year-old child initially presented with nonspecific and mild symptoms on the fifth day of the disease that responded to supportive care. Nonetheless, rapid deterioration ensued, characterized by worsening headaches, altered gait and speech, nausea, and vomiting, suggesting neurological involvement. Diagnostic evaluation can be challenging as bloodwork, imaging, and CSF-directed studies aimed to elucidate the underlying etiology may not yield conclusive results [5,10]. Some reasons include laboratory techniques might lack the sensitivity to detect the virus, the timing of sample collection might be poor as serum-conversion might be happening when serologic testing is performed, and the fact that there are no pathognomonic imaging findings of cerebral involvement, further complicating diagnosis [5,10,12].

Encephalitis-related VZV is reported to be more common in children than in adults [6,8,11], typically developing five to seven days after rash onset, with some studies suggesting that acyclovir may not be effective in the recovery of patients at this stage [12]. However, in our case, acyclovir was administered in an attempt to ameliorate the clinical condition by inhibiting the possible replication of a varicella virus. Even though there is no clear benefit of this antiviral in treating VZV meningitis or encephalitis [10], we aimed to treat his condition at a stage when additional tests were pending results. The two main differential diagnoses were a direct infection of the central nervous system by VZV or postinfectious encephalitis. The lack of early response to antiviral therapy and subsequent deterioration led to the stronger consideration of autoimmune encephalopathy. As a result, the decision was made to manage the condition with methylprednisolone in pulses. A five-day course of methylprednisolone was necessary before any improvement was clinically evident, as reported by other authors [13,14]. Even though there were no detectable autoantibodies or viruses in CSF, the progression of symptoms was consistent with an immune-mediated process. It is worth noting that there are documented cases of varicella-related neurological complications where VZV is undetectable [5,12]. Consequently, only a presumptive diagnosis of VZV-related encephalitis could be established.

Other diagnoses to consider would be encephalitis by other viruses. However, given the inconclusive laboratory results and clinical presentation (one week after symptom onset) and epidemiological context of VZV infection, other diagnoses were ruled out. Meningitis, either viral or bacterial, could be another infectious differential diagnosis; however, CSF analysis did not present results compatible with bacterial infection, and both viral and bacterial PCR tests in CSF were negative. Acute disseminated encephalomyelitis (ADEM) could be another diagnosis to consider: our patient presented in its age range (five to eight years), and there were multifocal neurological symptoms that could support the diagnosis. Clinically, this condition usually presents following an illness rather than during its course. Furthermore, radiologically, it is common to observe specific MRI findings supporting the diagnosis; however, such findings were absent in our case [15]. Clinical history did not raise the suspicion for toxic encephalitis as a diagnosis. An intracranial lesion could be suspected but was ruled out by brain imaging. As for another differential diagnosis, other autoimmune-mediated processes with specific antibodies could be the answer to our case. However, the autoantibodies analysis was negative for the targets assessed, and there were some that could not be measured. Therefore, although this condition did not fall within the typical spectrum of ADEM, the clinical presentation suggested an autoimmune process, leading us to base our therapeutic approach on immunosuppressive treatment.

Regarding the transient neurological deficits in our patient, even though they were only temporary, they persisted over a one-month period, and there was a need for physical and speech rehabilitation for a full recovery. Some countries recommend universal VZV vaccination to reduce disease severity, complications, and likelihood of hospitalization and transmission [2,16,17]. In Portugal, such a vaccine is not included in the national program of vaccinations [18]. Therefore, a diagnosis of varicella virus and its possible complications must always be present in the minds of healthcare professionals, particularly when a suggestive vesicular rash is or was recently present.

## Conclusions

This case underscores the importance of vigilance for potential complications in pediatric patients with primary varicella infection. It emphasizes the management challenges associated with neurological complications and that careful consideration of symptoms, epidemiological context, and a comprehensive evaluation through laboratory and imaging studies are needed to obtain an accurate diagnosis and adequate treatment.

It is not always possible to detect VZV or autoantibodies derived from an autoimmune response in the CSF. Therefore, when the presenting signs and evolution are strongly suggestive, as in our case, a diagnosis by presumption should be established. Even though, in our case, there was no observable response to antiviral therapy, prompt recognition of symptoms and early intervention with antiviral and/or immunomodulatory therapies, along with comprehensive rehabilitation strategies, are crucial in optimizing outcomes and mitigating long-term neurological sequelae, as well as for the patient's well-being.
